# Performance Analysis of a Deep Simple Recurrent Unit Recurrent Neural Network (SRU-RNN) in MEMS Gyroscope De-Noising

**DOI:** 10.3390/s18124471

**Published:** 2018-12-17

**Authors:** Changhui Jiang, Shuai Chen, Yuwei Chen, Yuming Bo, Lin Han, Jun Guo, Ziyi Feng, Hui Zhou

**Affiliations:** 1 School of Automation, Nanjing University of Science and Technology, Nanjing 210094, China; changhui.jiang1992@gmail.com (C.J.); byming@mail.njust.edu.cn (Y.B.); soochow_njust@sina.com (L.H.); guojun1136@163.com (J.G.); 2 Centre of Excellence in Laser Scanning Research, Finnish Geospatial Research Institute (FGI), Geodeetinrinne 2, FI-02431 Kirkkonummi, Finland; yuwei.chen@nls.fi (Y.C.); ziyi.feng@nls.fi (Z.F.); 3 Electronic Information School, Wuhan University, 129 Luoyu Road, Wuhan 430079, China; zhouhui@whu.edu.cn

**Keywords:** microelectromechanical systems, inertial measurement unit, simple recurrent unit, deep learning

## Abstract

Microelectromechanical System (MEMS) Inertial Measurement Unit (IMU) is popular in the community for constructing a navigation system, due to its small size and low power consumption. However, limited by the manufacturing technology, MEMS IMU experiences more complicated noises and errors. Thus, noise modeling and suppression is important for improving accuracy of the navigation system based on MEMS IMU. Motivated by this problem, in this paper, a deep learning method was introduced to MEMS gyroscope de-noising. Specifically, a recently popular Recurrent Neural Networks (RNN) variant Simple Recurrent Unit (SRU-RNN) was employed in MEMS gyroscope raw signals de-noising. A MEMS IMU MSI3200 from MT Microsystem Company was employed in the experiments for evaluating the proposed method. Following two problems were furtherly discussed and investigated: (1) the employed SRU with different training data length were compared to explore whether there was trade-off between the training data length and prediction performance; (2) Allan Variance was the most popular MEMS gyroscope analyzing method, and five basic parameters were employed to describe the performance of different grade MEMS gyroscope; among them, quantization noise, angle random walk, and bias instability were the major factors influencing the MEMS gyroscope accuracy, the compensation results of the three parameters for gyroscope were presented and compared. The results supported the following conclusions: (1) considering the computation brought from training dataset, the values of 500, 3000, and 3000 were individually sufficient for the three-axis gyroscopes to obtain a reliable and stable prediction performance; (2) among the parameters, the quantization noise, angle random walk, and bias instability performed 0.6%, 6.8%, and 12.5% improvement for X-axis gyroscope, 60.5%, 17.3%, and 34.1% improvement for Y-axis gyroscope, 11.3%, 22.7%, and 35.7% improvement for Z-axis gyroscope, and the corresponding attitude errors decreased by 19.2%, 82.1%, and 69.4%. The results surely demonstrated the effectiveness of the employed SRU in this application.

## 1. Introduction

Recently, Positioning, Navigation and Timing (PNT) information is essential for many applications, for example, smart mobile devices. Global Navigation Satellite System (GNSS) is the most widely used PNT provider, since it is easy to access the satellites signals all over the world [1,2,3]. A cheap receiver in a chip manner is sufficient for many applications, for instance, vehicle, smart phone, or the shared bicycle [1,2,3,4,5,6]. Narrowly speaking, GNSS refers to the United States of America (USA) Global Positioning System (GPS), European Galileo satellite navigation system, China BeiDou satellite navigation system (constructing), and Russia GLONASS navigation system [4,5,6], they aim to provide PNT service covering the earth for both civil and military users; broadly speaking, other regional satellite navigation systems are also included, for instance, Japan Quasi-Zenith Satellite System (QZSS) and Indian Regional Navigation Satellite System (IRNSS) [4,5,6]. With more and more satellites available in orbit broadcasting navigation signals, the user is able to make full use of more satellites signals in navigation solutions determination, which efficiently improves the reliability and continuity of the navigation solutions [4,5,6]. However, under some signal challenging or degraded environments, for instance, urban canyon and dense forests, the satellites signals are weak or even blocked, which makes it hard to output ideal or precise navigation solutions for the GNSS standalone navigation system [5,6,7,8]. Researchers have been devoted to overcome this problem and improve the performance under these signals degrading conditions [7,8,9,10]. Basically, there are two popular approaches in the navigation community. The first approach is to develop highly sensitive GNSS receiver for weak signals tracking, specifically high sensitive carrier tracking loop [11]. Abundant results have been published on this topic, including Vector Tracking Loop (VTL), Multiple Vector Tracking Loop (MVTL), and some other advanced carrier tracking loops design [11,12,13,14,15,16,17,18]. However, it is not sufficient for signal outage, no navigation solutions will be output, while GNSS signals are temporarily blocked by the buildings or something else [19,20]. 

The second approach is to integrate GNSS with other sensors, for instance, the Inertial Navigation System (INS), Visual navigation system, or LiDAR navigation system [21,22,23]. GNSS/INS integration system is the most popular for providing navigation solutions, including position, velocity, and attitude information. Inertial Navigation System (INS) is a self-contained navigation system which generates continuous navigation solutions by processing the measurements from Inertial Measurement Unit (IMU). IMU usually contains three orthogonal gyroscope and three orthogonal accelerometers, in which the gyroscope measures the angle rate and the accelerometer collects the acceleration motion [24,25,26]. When compared with GNSS, the navigation solutions updating frequency is higher, while the INS is usually 400 Hz or 200 Hz, and the updating frequency of the GNSS solutions is commonly 1–10 Hz [21,22,23,24,25]. However, the GNSS is capable of providing precise location with well accessible signals, while the errors of the INS diverges over time, due to the various unavoidable noises contained in the raw measurements or signals of the employed gyroscopes and accelerometers. The advantage of the GNSS/INS integration navigation system is that the INS can still provide the navigation solutions, while the GNSS fails to generate navigation solutions. Therefore, it is of great value to improve the INS accuracy during GNSS signal outage. 

Conventional fiber optic or laser gyroscope is of large size with high cost and it is not proper or suitable for certain applications, especially handheld or portable devices. Microelectromechanical systems (MEMS) IMU is increasingly popular recently, which is manufactured using MEMS technology. MEMS IMU has much smaller size and lower cost, and it has been widely used in mobile or handheld devices, vehicle navigation and weapons guidance [26,27,28,29,30,31,32,33,34,35]. However, compared with the highly accurate laser or fiber optic IMU, MEMS IMU usually experiences more complicated noises [26,27,28,29,30,31,32]. Scholars are always devoted to model the noises and compensate the divergence errors to improve the accuracy of MEMS IMU based navigation system, which can broaden the MEMS IMU applications furtherly [27,28,29,30,31,32,33,34,35]. As illustrated in previous papers, the MEMS IMU noise modelling or analysis method can be classified to the statistical method and Artificial Intelligence (AI) method [33,34,35,36]. The statistical methods include Auto Regressive Moving Average (ARMA) and Allan Variance (AV), especially the Allan Method has been widely used to analyze and describe the composition of the gyroscope or the accelerometer noise contained in the output raw signals [33,34,35,36]. Generally, the five basic description parameters are termed as: quantization noise, angle random walk, bias instability, rate random walk, and rate ramp [33,34,35,36]. The other method is the Artificial Intelligence, which refers to Support Vector Machine (SVM) and Neural Networks (NN). SVM and various Neural Networks are employed in MEMS IMU de-noising and they have been evaluated by many researchers [33,34,35,36]. 

For both statistical or AI methods, gyroscope or accelerometer raw signals are treated as time series, and a model is described or learned to compensate the errors caused by the noises. Performance of the statistical method is limited by the fixed model parameters, and the traditional AI method has restricted learning capacity which is determined by the structure and working principles [26,27,28,29,30,31,32,33,34,35,36]. Recently, Deep Learning (DL) gains a boom in various applications, which has a better learning capacity than conventional SVM or neural networks [37,38]. Deep Recurrent Neural Networks (RNN) is specifically for processing time series data and has been demonstrated effectively in this kind of application [38,39,40]. In our previous paper, a Long Short Term Memory Unit (LSTM) (a variant of RNN) was evaluated in MEMS gyroscope de-noising, and experiments included a comparison of LSTM-RNN with ARMA, single-layer LSTM, and multi-layer LSTM [38]. Limitations of the proposed algorithms exposed in the paper were listed as following:

(1) Training data length is fixed and not long enough, it might be meaningful to explore the influence of the training data length on the deep RNN performance; and,
(2) Only Standard Deviation of the de-noised signals were presented and compared, but no detailed or further analysis of compensation, which could be a support of selecting proper neural networks for each MEMS IMU.

In this paper, an advanced variant of RNN Simple Recurrent Unit (SRU) was investigated in MEMS IMU based navigation system for accuracy improvement. When compared with LSTM, SRU has less simple structure with faster training speed, specifically; the SRU has less parameter that need to be determined during training procedure [39,40]. We think the extensions or contributions of this paper are as follows:

(1) Influence of training dataset length on the SRU-RNN prediction were investigated and explored, it might be meaningful for reducing computation load;
(2) Compensation degrees of the major noise parameters describing MEMS IMU performance were presented and compared, which might support the selection of proper or suitable RNN variants for MEMS IMU de-noising;
(3) SRU was firstly employed in this application; the results could be compared with LSTM presented in our previous paper for selecting proper RNN in MEMS IMU de-noising.

Reminder of this paper is organized as: (1) the second section gives the basic mathematical equations and the information flow of the popular SRU-RNN; (2) in the next section, the experiments results and comparisons are presented to support the conclusions; (3) final sections include the conclusion, discussion, and reference. 

## 2. Method

In this section, the basic structure and mathematical equations of the Simple Recurrent Unit Recurrent Neural Networks (SRU-RNN) are introduced and described. This section is divided into two parts: (1) the architecture and mathematical equations are listed in Section 2.1; (2) the implementation and the deep SRU-RNN working flow are given in detail in Section 2.2.

### 2.1. Simple Recurrent Unit

**Figure 1:**
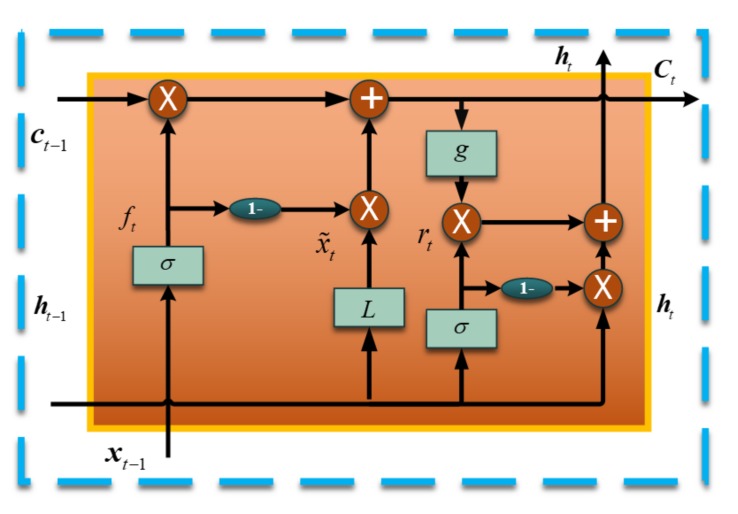
Basic structure of the Simple Recurrent Unit (SRU) Working flow.

Simple Recurrent Unit (SRU) is the recently proposed variant of RNN, and the SRU has a more concise structure for accelerating the training procedure [39]. When compared with other RNN variants, for instance, Long Short Term Unit (LSTM) and Grated Recurrent Unit (GRU), the SRU has faster training speed that is brought by its unique structure [39,40]. Figure 1 shows the basic architecture of the employed SRU, and it is constructed based on the “gate” structure, which also is composed of LSTM and GRU. In a single layer SRU illustrated as Figure 1, it is usually has two essential components, which are termed as “light recurrence” and “high network”. The light recurrence component reads the input vector $x_{t}$ and calculates the sequence of the state $c_{t}$. This module captures the sequential information [39,40]. The light recurrent procedure can be summarized as the following Equations (1)–(3):

(1) $${\tilde{x}}_{t}=Wx_{t}$$

(2) $$f_{t}=\sigma \left(W_{f}x_{t}+b_{f}\right)$$

(3) $$c_{t}=f_{t}⊙c_{t-1}+\left(1-f_{t}\right)⊙\left(Wx_{t}\right)$$

Where, $W_{f}$, W, and $b_{f}$ are the parameter matrices, which will be determined through the training process. σ(⋅) is a sigmoid function and the $f_{t}$ is output of this function ranging from 0 to 1. ⊙ is the point-wise multiplication operation. 

As illustrated in Figure 1, the $f_{t}$ controls the information flow and the current state $c_{t}$ is determined by adaptively averaging the previous state $c_{t-1}$ and the current input vector according to $f_{t}$. Especially, the SRU is different in using the previous state $c_{t-1}$. Traditionally, each dimension of $c_{t}$ and $f_{t}$ depends on all entries of $c_{t-1}$, and the computation has to wait until $c_{t-1}$ is fully computed. In the SRU, a point-wise multiplication ⊙ is employed to hence the parallelization (seen as Equations (1) and (2)).

The second component of SRU is the highway network, which is employed to facilitate gradient-based training of deep networks [39,40]. A reset ‘gate’ $r_{t}$ is used to combine the input vector $x_{t}$ and the current state $c_{t}$ from the light recurrence [39,40]. The highway network equations are as following:

(4) $$r_{t}=\sigma \left(W_{t}x_{t}+b_{r}\right)$$

(5) $$h_{t}=r_{t}⊙g\left(c_{t}\right)+\left(1-r_{t}\right)⊙x_{t}$$

Where, $W_{r}$ and $b_{r}$ are also the parameters learned by the training procedure. Especially, $\left(1-r_{t}\right)⊙x_{t}$ is a skip connection allowing the gradient to directly propagate to the previous layer, which has shown to improve the scalability [39,40]. 

### 2.2. Deep SRU-RNN Implementation

**Figure 2:**
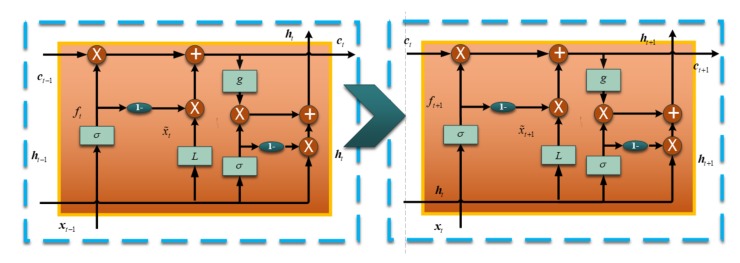
Working flow of two Simple Recurrent Unit–Recurrent Neural Networks.

**Figure 3:**
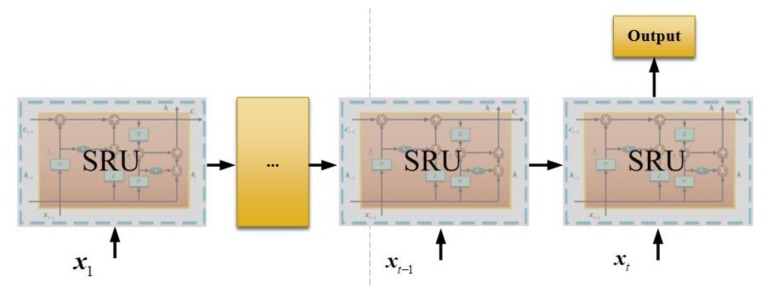
Basic structure of Deep Simple Recurrent Unit–Recurrent Neural Networks Training.

Figure 1 is just a single-layer SRU unit and Figure 2 shows the information flow of the two SRU units. The cell state is conveyed to the next SRU unit and the next SRU combines it with the input vector to decide the outputs. Basically, a sequence SRU decides the output together, and the structure is shown in Figure 3. A large dataset is necessary for training the deep SRU-RNN, and the parameters in each SRU (shown in Equations (1)–(5)) are determined or learned during the training procedure.

## 3. Experiments

**Figure 4:**
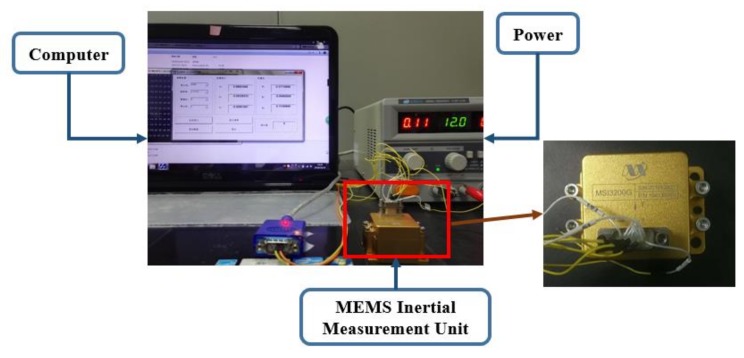
Gyroscope and Accelerometer raw signals collecting.

With the aim of evaluating and verifying the proposed method, a MEMS IMU MSI3200manufactured by MT Microsystems Company (Hebei, China) is employed in the following experiments [41]. Since attitude errors play an important role in position accuracy, only gyroscope de-noising results are presented and analyzed in this paper. A dataset with approximately 10 min time length is collected and the data collecting set up is shown in Figure 4. Several devices are employed in the operation including the MEMS IMU, a laptop, power supply and some cables. The MEMS IMU works at 12 volts and a laptop is employed to store the data. The IMU is composed of three-orthogonal gyroscopes and three-orthogonal accelerometers, and Table 1 lists the parameters of the employed MEMS IMU in detail. During the raw signal collecting, the MEMS IMU is placed on the table statically and the sampling frequency is set to 400 Hz here. The gyroscope output unit is degree/s and the accelerometer output unit is g (1 g = 9.8 m/s^2^). Furtherly, according to the IMU setting on table, specifically, the X-axis gyroscope measures the pitch angle, the Y-axis measures the roll angle, and the Z-axis measures the yaw angle. 

### 3.1. Traing Data Length Analysis

**Figure 5:**
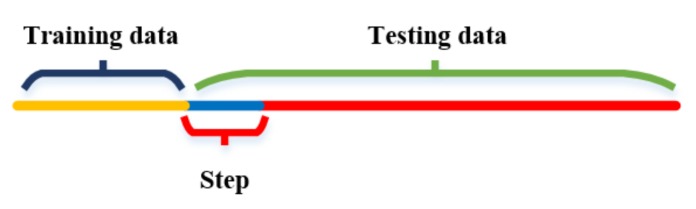
Data structure.

In this sub-section, the influence of the training length on the SRU-RNN is discussed. Parameters of the employed SRU-RNN are listed in Table 2. Specifically, the training epoch is fixed as 100, while the length of the input data varies. The structure of the training data, input data length termed as step and testing data are explained in Figure 5. The yellow part of the line represents the training data, the middle blue part is the “step”, and the testing data includes the blue and red parts of the line. Since longer training data means more computation, and thus there might be a trade-off between training data length and prediction accuracy. Table 2 also lists the other specifications of the SRU-RNN employed. The learning rate is set to 0.01, and the hidden unit amount is 1. Moreover, the input data size or step is set 10. 

**Figure 6:**
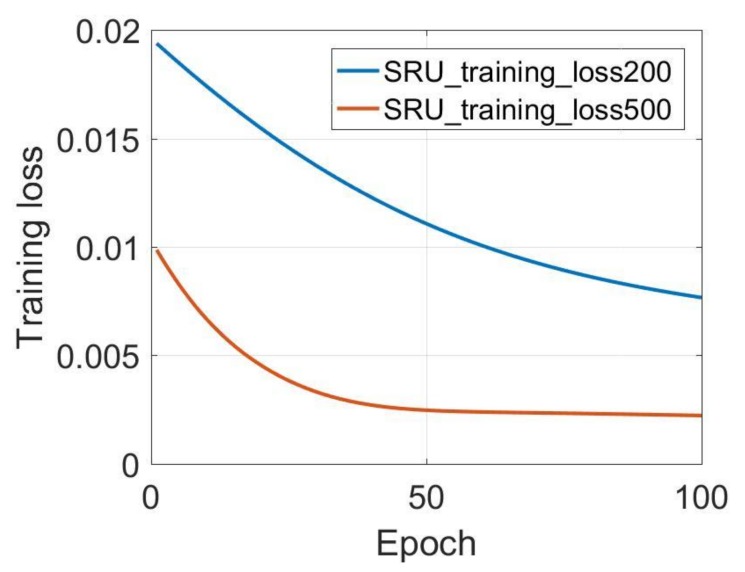
X-axis gyroscope training loss comparison.

Table 3 lists the prediction results of different training data length for the three-axis gyroscope, while the input data size and testing data size are fixed. The training data size varies from 200 to 10,000. For the X-axis gyroscope, the standard deviation (STD) values varying from 0.054 to 0.062 with the software running time varies from 57.8 s to 77.97 s. Figure 6 shows the SRU-RNN training loss comparison between data length of 200 and 500. The blue line represents the SRU training loss, with 200 training data length. The red line represents the SRU-RNN training loss with 500 training data length. From the Figure 6, it can be seen that SRU with a training date length of 200 does not converge within the set epoch of 100. However, with the 500 input data length for training, the SRU-RNN is able to converge within 100 training epoch. 

**Figure 7:**
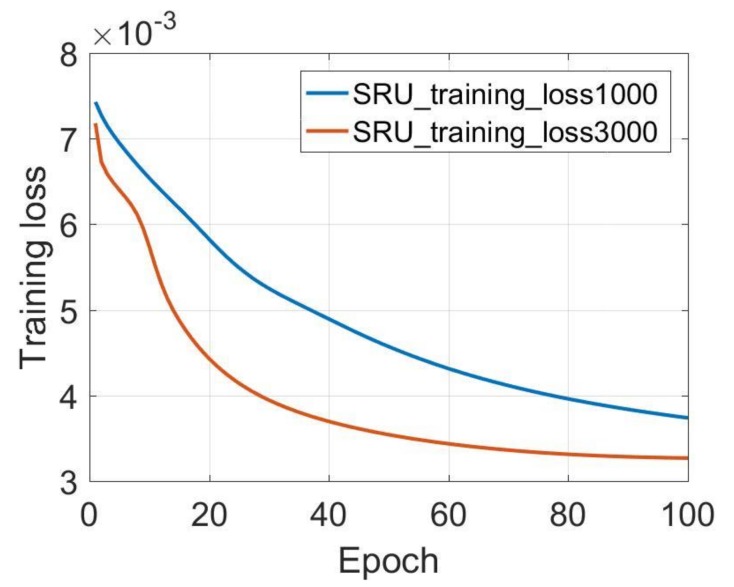
Y-axis gyroscope training loss comparison.

**Figure 8:**
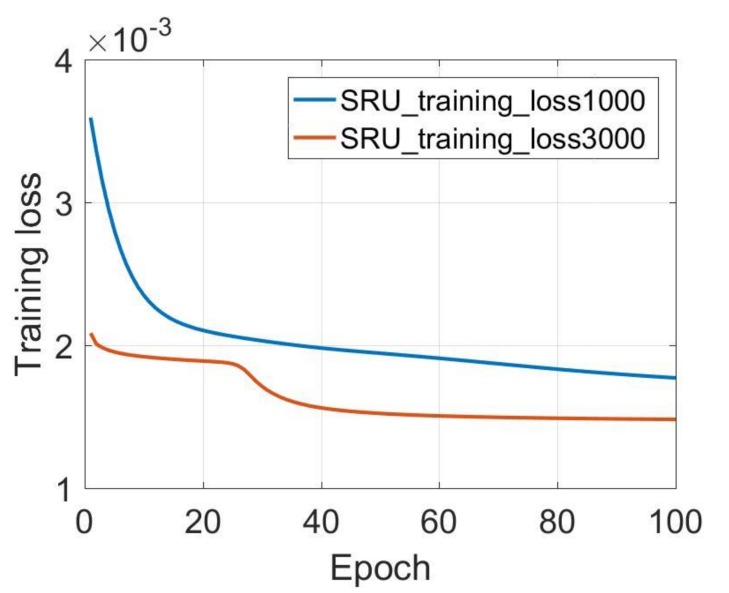
Z-axis gyroscope training loss comparison.

In addition, the Y-axis and Z-axis gyroscopes results are also listed in Table 3. Similarly, the SRU-RNN is unable to converge when the data length is not sufficient with the 100 training epoch. Figure 7 and Figure 8 show the training loss comparisons. It can be seen that SRU-RNN with 3000 training data length converge, while the SRU-RNN with 1000 training data length is unable to converge within the set training epoch values. In theory, more training data will lead to slower convergence speed. However, from the results, the SRU-RNN with more training data converges faster. SRU-RNN is a deep recurrent neural network depending on the memory. Under this condition, while the training data is of small length, it is not sufficient for this SRU-RNN training and learning of the model. Thus, the SRU-RNN is able to converge with sufficient training data length. In aspects of the standard deviation (STD) of the de-noised testing data, the STD values keeps almost the same, which might demonstrate that the SRU-RNN has been well trained. Therefore, for the X-axis gyroscope (Figure 6), 500 is sufficient, however, for Y-axis and Z-axis gyroscopes (Figure 7 and Figure 8), 3000 is sufficient. The difference between the three gyroscopes might be caused by the MEMS manufacturing technology, which leads to the difference in the three-axis gyroscopes signals characteristics. 

### 3.2. Different Parameters Compensation Analysis

**Figure 9:**
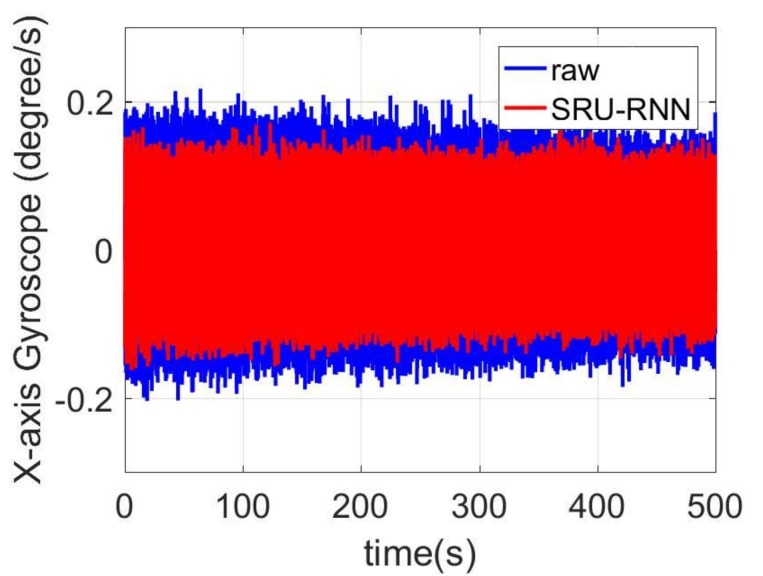
De-noised and raw signals comparison for X-axis gyroscope.

**Figure 10:**
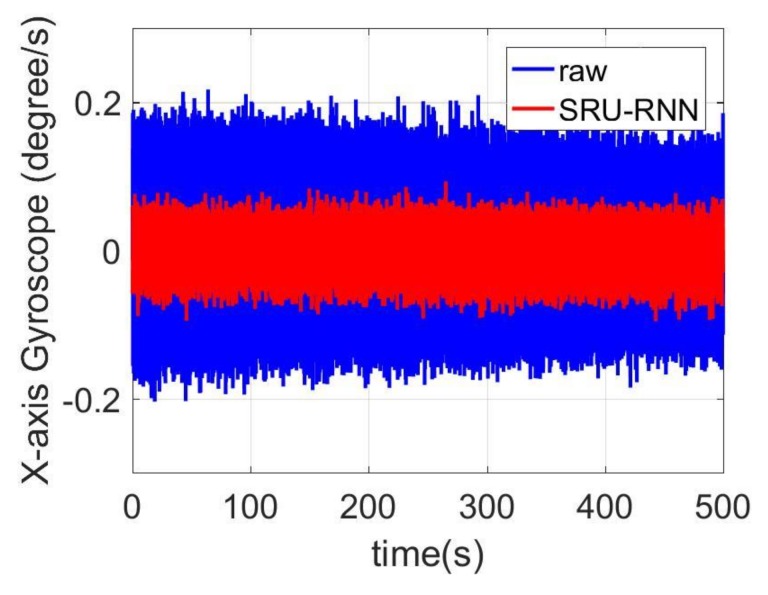
De-noised and raw signals comparison for Y-axis gyroscope.

**Figure 11:**
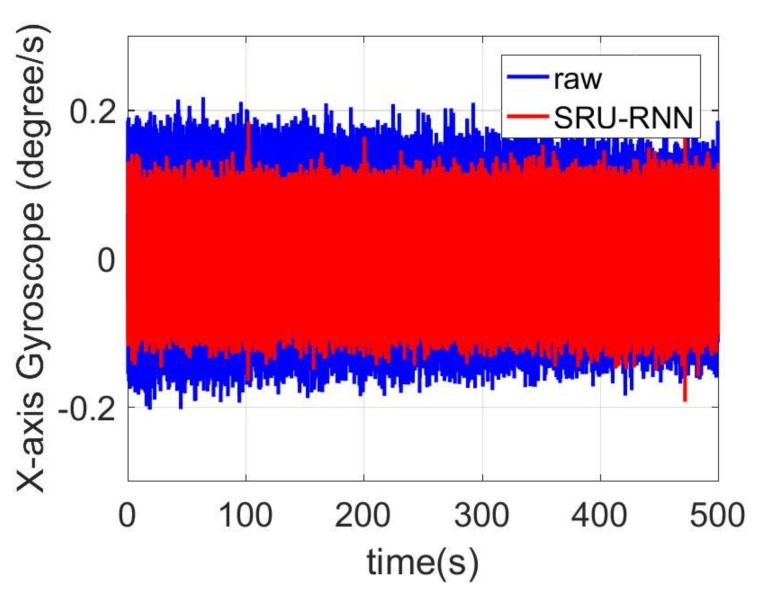
De-noised and raw signals comparison for Z-axis gyroscope.

**Figure 12:**
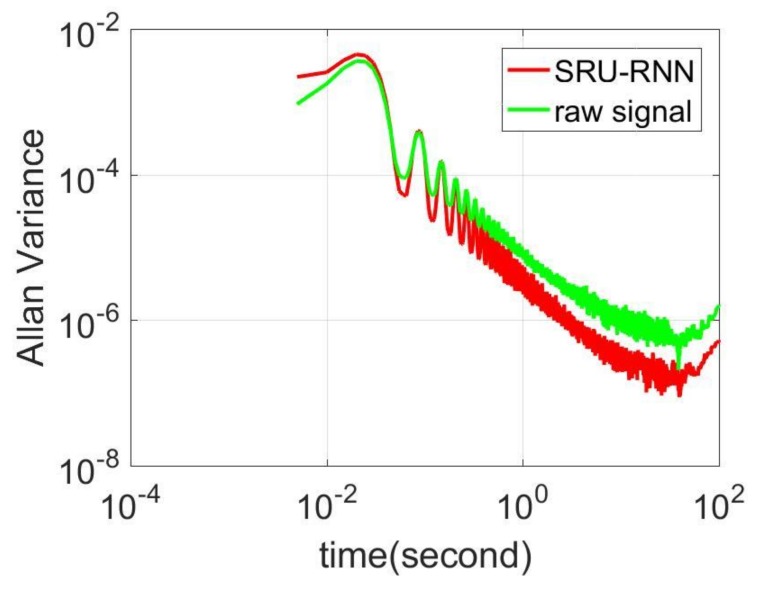
Allan variance comparison between de-noised and raw signals for X-axis gyroscope.

**Figure 13:**
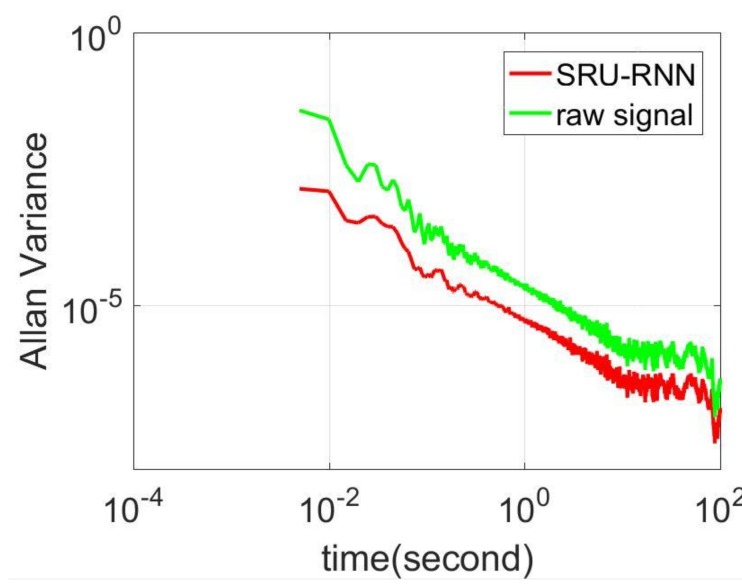
Allan variance comparison between de-noised and raw signals for Y-axis gyroscope.

**Figure 14:**
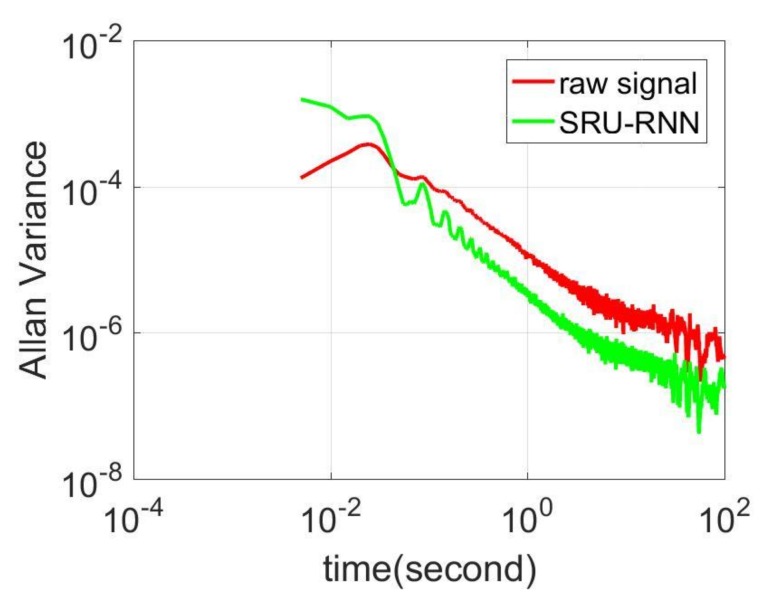
Allan variance comparison between de-noised and raw signals for Z-axis gyroscope.

In Section 3.1, the influence of the training data length on the training or prediction results were analyzed. As aforementioned in the introduction section, quantization noise, angle random walk, and bias instability are the major index for describing the MEMS IMU performance. Thus, Figure 9, Figure 10 and Figure 11 shows the comparison of the de-noised and raw signals, Figure 12, Figure 13 and Figure 14 presented the Allan Variance comparison results, and Table 4, Table 5 and Table 6 show the comparison of the parameters between raw signals and de-noised signals. For the X-gyroscope, the quantization noise has a minor improvement, while the angle random walk and bias instability have a 6.8% and 12.5% improvement, respectively. The Y-axis gyroscope has a 60.5% improvement in quantization parameters and 17.3% and 34.1% in angle random work and bias instability, respectively. The rest Z-axis gyroscope has an improvement of 11.3%, 22.7%, and 35.7% in parameters of quantization noise, angle random walk, and bias instability individually. The attitude errors are listed in Table 7, the roll, pitch, and yaw angles errors decreased by 19.2%, 82.1%, and 69.4% individually. In this experiment, the X-axis gyroscope measured the pitch angle, the Y-axis gyroscope measures the roll angle, and the Z-axis gyroscope measures the yaw angles. These attitude angles were calculated based on the quaternion algorithms, and more detailed could be found in the reference [42]. Since the IMU was placed statically in a table, the initial values were all set to zero. 

**Figure 15:**
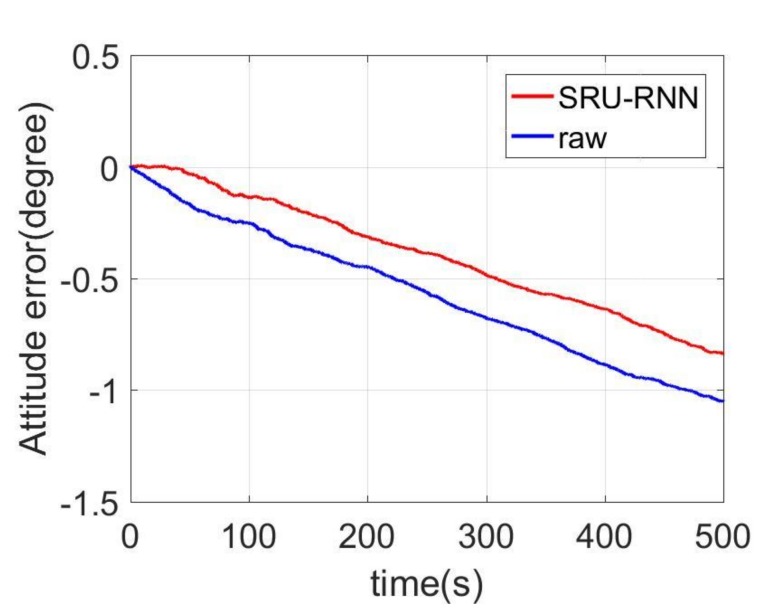
Pitch angles.

**Figure 16:**
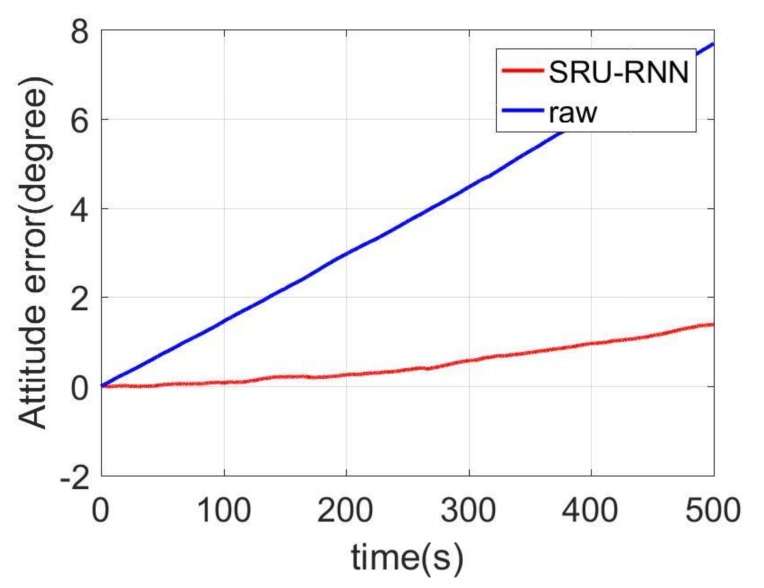
Roll angle errors comparison.

**Figure 17:**
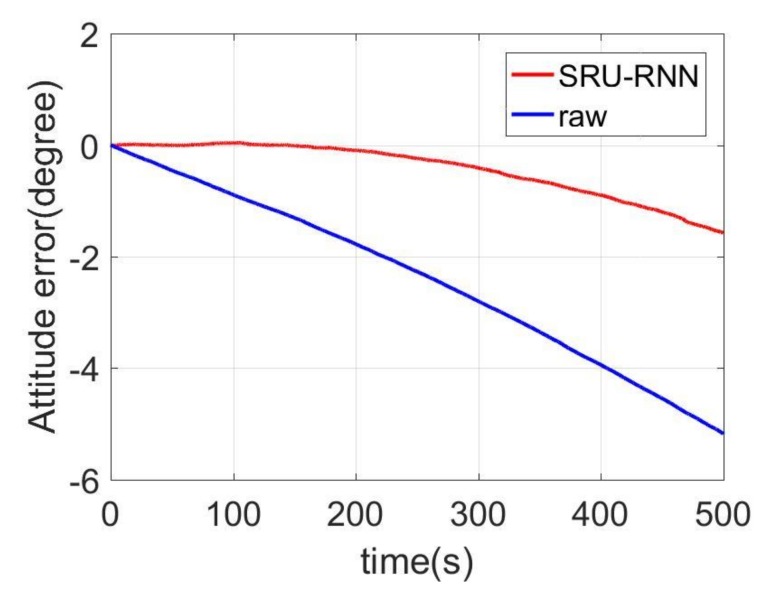
Yaw angle errors comparison.

As aforementioned in Section 3, the X-axis gyroscope measures the pitch angle, the Y-axis measures the roll angle, and the Z-axis measures the yaw angle. Figure 15, Figure 16 and Figure 17 shows the attitude errors. The roll angle performs the least decrease, which is reflected from the corresponding X-axis gyroscope analysis. For the raw and pitch angles, they have an improvement of 82.1% and 69.4% with the 500 s time length data. The difference between raw angle and pitch angle is caused by that the Y-axis gyroscope has smaller quantization noise. The Y-axis and Z-axis gyroscope have similar angle random walk and bias instability (listed in Table 5 and Table 6). However, the Y-axis has a 60.5% decrease in quantization noise, while the Z-axis gyroscope just has 11.3% improvement.

## 4. Conclusions

This paper investigated a deep Simple Recurrent Unit Recurrent Neural Networks (SRU-RNN) based MEMS gyroscope de-noising method, from the experimental results, these conclusions were obtained:

(1) There was a trade-off between the training data length and the de-noising performance, for the employed Inertial Measurement Unit, 500, 3000, and 3000 was sufficient for learning the model with set 100 training epoch;
(2) Among the major three Inertial Measurement Unit errors describing parameters, there was no regular pattern for the compensation degree of the parameters;
(3) The three-axis attitude had an improvement of 19.2%, 82.1%, and 69.4%, and which is consistent with the analysis from the three gyroscope signals. The results demonstrated the effectiveness of the proposed SRU-RNN method.

However, there were following limitations of this paper:

(1) In the experiments, the SRU-RNN was trained with fixed parameters including the learning rate and batch size. Parameters optimization might improve the performance of the SRU-RNN in this application. Some optimization methods are available in the AI community;
(2) As aforementioned, the SRU-RNN is single layer, and actually a multi-layer SRU-RNN might improve the performance of the SRU-RNN;
(3) In the experiments, limited by the lab equipment, only the static dataset was collected and employed.

Future work will include: (1) further analysis in signals characteristics is necessary for exploring what causes the difference in training data length; (2) comparing SRU-RNN, LSTM-RNN, and some other variants of RNN using same MEMS IMU dataset, finding suitable RNN for MEMS gyroscope and accelerometer raw signals de-noising; (3) dynamic or field testing dataset will be employed for furtherly investigated the deep learning method in application of MEMS IMU noises modeling; and, (4) it will be great significance of embedding a deep learning module in MEMS IMU, a feasibility study will be conducted in a GNSS/MEMS IMU integrated navigation system for evaluating the performance in GPS signal outage of 60 s.

## Figures and Tables

**Table 1:** Specifications of MSI3200 IMU.

**MEMS IMU**	Gyroscope	Range	±300°/s
		Bias instability (1σ)	≤10°/h
		Bias instability (Allan)	≤2°/h
		Angle random walk	≤0.15°/$\sqrt{h}$
	Accelerometer	range	±15 g
		bias instability (1σ)	0.5 mg
		bias repeatability (Allan)	0.5 mg
	Power consumption	1.5 W	
	Weight	250 g	
	Size	70 mm×54 mm×39 mm	
	Sampling rate	400 Hz	

**Table 2:** Specifications of Simple Recurrent Unit Recurrent Neural Networks (SRU-RNN).

Batch size	128
Training epoch	100
Learning rate	0.01
Hidden unit amount	1

**Table 3:** Training results of the three-axis gyroscope.

	*X*		*Y*		*Z*	
Training Data Length	STD (Degree/s)	Time (Second)	STD (Degree/s)	Time (Second)	STD (Degree/s)	Time (Second)
10,000	0.062	77.0	0.057	121.5	0.025	86.9
3000	0.054	61.3	0.054	99.0	0.023	82.3
1000	0.055	57.8	/	/	/	/
500	0.055	60.7	/	/	/	/
200	/	57.7	/	/	/	/
/	0.073	/	0.082	/	0.045	/

**Table 4:** Parameters of X-axis gyroscope results.

	X		
	Raw	SRU-RNN	Percentage
quantization noise (deg/$\sqrt{h}$)	0.15	0.15	0.6%
Angle random walk (deg/$\sqrt{h}$)	0.44	0.41	6.8%
Bias instability (deg/h)	2.48	2.17	12.5%

**Table 5:** Parameters of Y-axis gyroscope results.

	Y		
	Raw	SRU-RNN	Percentage
quantization noise (deg/$\sqrt{h}$)	1.0	0.40	60.5%
Angle random walk (deg/$\sqrt{h}$)	0.23	0.19	17.3%
Bias instability (deg/h)	1.29	0.85	34.1%

**Table 6:** Parameters of Z-axis gyroscope results.

	Z		
	Raw	SRU-RNN	Percentage
quantization noise (deg/$\sqrt{h}$)	0.62	0.55	11.3%
Angle random walk (deg/$\sqrt{h}$)	0.22	0.17	22.7%
Bias instability (deg/h)	1.12	0.72	35.7%

**Table 7:** Attitude results comparison.

	Attitude		
	Raw	SRU	Percentage
Pitch/(degree)	−1.04	−0.84	19.2%
Roll/(degree)	7.69	1.38	82.1%
Yaw/(degree)	−5.18	−1.58	69.4%
